# Two Novel *katG* Mutations Conferring Isoniazid Resistance in *Mycobacterium tuberculosis*

**DOI:** 10.3389/fmicb.2020.01644

**Published:** 2020-07-15

**Authors:** Li-Yu Hsu, Li-Yin Lai, Pei-Fang Hsieh, Tzu-Lung Lin, Wan-Hsuan Lin, Hsing-Yuan Tasi, Wei-Ting Lee, Ruwen Jou, Jin-Town Wang

**Affiliations:** ^1^ Department of Microbiology, National Taiwan University College of Medicine, Taipei, Taiwan; ^2^ Tuberculosis Research Center, Taiwan Centers for Disease Control, Taipei, Taiwan; ^3^ Diagnostics and Vaccine Center, Taiwan Centers for Disease Control, Taipei, Taiwan; ^4^ Department of Internal Medicine, National Taiwan University Hospital, Taipei, Taiwan

**Keywords:** *Mycobacterium tuberculosis*, drug resistance, isoniazid, mutation, *katG*

## Abstract

Tuberculosis (TB), an infectious disease caused by *Mycobacterium tuberculosis*, is among the top 10 leading causes of death worldwide. The treatment course for TB is challenging; it requires antibiotic administration for at least 6 months, and bacterial drug resistance makes treatment even more difficult. Understanding the mechanisms of resistance is important for improving treatment. To investigate new mechanisms of isoniazid (INH) resistance, we obtained three INH-resistant (INH-R) *M. tuberculosis* clinical isolates collected by the Taiwan Centers for Disease Control (TCDC) and sequenced genes known to harbor INH resistance-conferring mutations. Then, the relationship between the mutations and INH resistance of these three INH-R isolates was investigated. Sequencing of the INH-R isolates identified three novel *katG* mutations resulting in R146P, W341R, and L398P KatG proteins, respectively. To investigate the correlation between the observed INH-R phenotypes of the clinical isolates and these *katG* mutations, wild-type *katG* from H37Rv was expressed on a plasmid (pMN437-*katG*) in the isolates, and their susceptibilities to INH were determined. The plasmid expressing H37Rv *katG* restored INH susceptibility in the two INH-R isolates encoding the W341R KatG and L398P KatG proteins. In contrast, no phenotypic change was observed in the KatG R146P isolate harboring pMN437-*katG*. H37Rv isogenic mutant with W341R KatG or L398P KatG was further generated. Both showed resistant to INH. In conclusion, W341R KatG and L398P KatG conferred resistance to INH in *M. tuberculosis*, whereas R146P KatG did not affect the INH susceptibility of *M. tuberculosis*.

## Introduction

According to the Global Tuberculosis Report published by the World Health Organization (WHO), tuberculosis (TB), the airborne infectious disease caused by *Mycobacterium tuberculosis* (Cambau and Drancourt, 2014), is one of the top 10 causes of death worldwide, and thus remains a major global public health problem (WHO, 2019). The emergence of drug-resistant TB has made the need for improvements in diagnostic accuracy and successful treatment even more urgent, as both are major challenges in TB control and key causes of its high mortality rate (Nguyen et al., 2019).

Since the 1940s, several drugs have been developed for the treatment of TB (Sotgiu et al., 2015; Kurz et al., 2016). These drugs can be classified as first-line anti-TB drugs, including the isoniazid (INH), rifampicin, pyrazinamide, and ethambutol, as well as other second-line drugs, which are used in cases of treatment failure (Rendon et al., 2016). The first-line anti-TB drug, INH, which was initially shown to have anti-TB activity in 1952 (Fox, 1952), is suitable for treatment when *M. tuberculosis* is replicating (Chakraborty and Rhee, 2015). INH is a prodrug that is activated by the catalase-peroxidase KatG. The metabolites produced then react with nicotinamide adenine dinucleotide (NAD^+^), and binding of the INH-NAD adduct to the NADH-dependent enoyl-ACP reductase InhA. InhA inhibits mycolic acid formation and cell wall synthesis in *M. tuberculosis*, leading to cell death (Vilchèze and Jacobs, 2014; Chakraborty and Rhee, 2015; Islam et al., 2017).

Most INH-resistant (INH-R) strains harbor mutations in genes associated with cell wall synthesis, the *katG* gene (Somoskovi et al., 2001; Laurenzo and Mousa, 2011), the *inhA* gene and its promoter (Zhang et al., 1992; Nguyen et al., 2019), or the *oxyR*-*ahpC* region (Sreevatsan et al., 1997; Lempens et al., 2018). Articles suggest that *katG* deletion mutants have higher INH resistance than strains with mutations in *inhA* or its promoter (Somoskovi et al., 2001; Lempens et al., 2018). In addition, upregulation of INH inactivators or efflux pumps was involved in INH resistance (Vilchèze and Jacobs, 2014; Unissa et al., 2016).

In this work, three INH-R clinical isolates with minimum inhibitory concentrations (MICs) ≥64 mg/L were found to have novel *katG* mutations that were not previously reported to confer INH resistance. The aim of this study was to examine whether the amino acid changes encoded by the *katG* mutations in these high-level INH-R clinical isolates are determinants of INH resistance. We compared the INH susceptibility of these isolates to those of isolates expressing the H37Rv KatG protein, and then recreated these point mutations in H37Rv to confirm the relationship between the *katG* mutations and INH resistance.

## Materials and Methods

### Ethics Statement

According to the Taiwan Communicable Disease Control Act, TB is one of the notifiable diseases, and specimen collection for laboratory testing is mandatory. This study did not require ethics approval, and participant consent was not required.

### Bacterial Strains and Culture Conditions

The bacterial strains used in this study are listed in Supplementary Table S1. All the experiments for *M. tuberculosis* strains were carried out at a BSL-3 laboratory in National Taiwan University College of Medicine, Taiwan, following institutional biosafety procedures. The INH-R *M. tuberculosis* isolates identified by clinical TB laboratories of Taiwan were sent to the Reference laboratory of Mycobacteriology at the Taiwan Centers for Disease Control (TCDC) for confirmation. Reference strain *M. tuberculosis* H37Rv, H37Rv-derived isogenic mutants, and the clinical INH-R isolates were grown in Middlebrook 7H9 liquid medium (BD Difco, Sparks, MD, USA) containing 10% oleic acid/albumin/dextrose/catalase (OADC; BD Difco, Sparks, MD, USA), 0.5% glycerol, and 0.05% Tween-80 or Middlebrook 7H11 solid agar (BD Difco, Sparks, MD, USA) containing 10% OADC and 0.5% glycerol at 37°C. *Escherichia coli* DH10B, for plasmid construction, was grown in Luria-Bertani (LB) medium (Bio Basic, Toronto, Canada) at 37°C. The following were added to medium as needed for selection: 50 mg/L hygromycin (BioShop, Ontario, Canada), 100 mg/L X-gal, and 4% sucrose for *M. tuberculosis* and 100 mg/L hygromycin (BioShop, Ontario, Canada) for *E. coli*.

### Screening and Sequencing of INH-R Clinical Isolates

The MIC of INH-R *M. tuberculosis* isolates was screened and determined using the Sensititre™ MYCOTB MIC Plate (Trek Diagnostic Systems, OH, USA) according to the manufacturer’s instructions. The ranges of drug concentrations were 0.03–4 mg/L for INH. The bacterial solution was adjusted to turbidity at a McFarland standard of 0.5 and then added to the Sensititre™ MYCOTB MIC Plate before the plate was covered with the adhesive plastic seal. After incubation at 37°C for 14 or 21 days, the results were recorded using the Sensititre™ Vizion™ Digital MIC Viewing System.

The primers used to sequences *katG*, *inhA*, *oxyR*, and *aphC* are listed in Supplementary Table S2. The polymerase chain reaction (PCR) cycling conditions were previously described (Jou et al., 2019). The sequences of *katG* in INH-R strains CDC-A, CDC-B, and CDC-C had been submitted to NCBI (GenBank accession numbers: MT572851, MT572852, and MT572853).

### KatG Expression Constructs

The primer and plasmids used in this study are listed in Supplementary Tables S2, S3. To express wild-type *katG* in the INH-R clinical strains, a *katG* expression plasmid, pMN437-*katG*, was generated by ligating the *katG* gene from *M. tuberculosis* H37Rv to pMN437 (Song et al., 2008; Steinhauer et al., 2010), which was linearized *via* reverse PCR to remove the *gfp* gene. The pMN437 and pMN437-*katG* plasmids were transformed into competent *M. tuberculosis* cells by electroporation at 2,500 V, 1,000 Ω, and 25 μF as previously described (Larsen et al., 2007).

### Mutagenesis of *katG* in H37Rv

To replace the *katG* gene in H37Rv with the mutant genes in the INH-R clinical isolates, fragments of the mutated *katG* genes in clinical strains CDC-A and CDC-B were amplified and ligated into the pGOAL19 plasmid at the *Sca*I site. The primers used are listed in Supplementary Table S2. The pGOAL19 plasmid is a suicide plasmid that lacks a mycobacterial origin for plasmid replication (Parish and Stoker, 2000). When the pGOAL19 recombinant plasmid (pGOAL19-*katG* W341R R463L or pGOAL19-*katG* L398P R463L) was transformed to the H37Rv strain, a two-step homologous recombination has *occurred* between the plasmid and the genome results in replacement of the H37Rv gene with the mutant gene. The H37Rv isogenic mutants were confirmed by PCR and sequencing.

### INH Susceptibility Tests

To evaluate the effect of H37Rv KatG expression in INH-R isolates and the impact of KatG from the INH-R isolates in H37Rv isogenic mutants, the INH susceptibility was assessed by the agar dilution assay. Briefly, 5 μl of a 4 × 10^6^ CFU/ml bacterial suspension (equivalent to 2 × 10^4^ cfu) was spotted on Middlebrook 7H11 agar plates containing 10% OADC, 0.5% glycerol, and serial diluted INH (Sigma, St. Louis, MO, USA) concentrations of 0, 0.2, 1, 4, 16, and 64 mg/L and incubated at 37°C. The results were recorded after 3 weeks of incubation. Resistance was defined as colonies growing in the presence of the critical concentrations of 0.2 mg/L INH, according to the CLSI guidelines (Clinical and Laboratory Standards Institute, 2011). All reported MICs were represented from three independent experiments. For the H37Rv KatG expression, H37Rv/pMN437 was used as the control strain, and 50 mg/L hygromycin was added to the agar plates to maintain the transformed plasmids. To compare the INH susceptibility of KatG from the clinical isolates in H37Rv isogenic mutants, H37Rv was used as the control.

## Results

### Three INH-R Clinical Isolates Harbor Novel Mutations in *katG*

**Figure 1:**
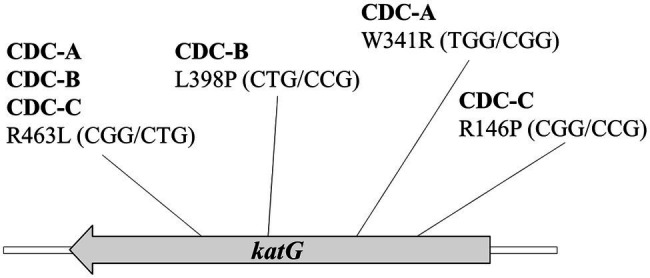
The *katG* sequence results of three high INH resistance strains CDC-A, CDC-B, and CDC-C shows that all strains encode a mutation at R463L (CGG/CTG) and another mutation at W341R (TGG/CGG), L398P (CTG/ CCG), and R146P (CGG/CCG), respectively.

**Figure 2:**
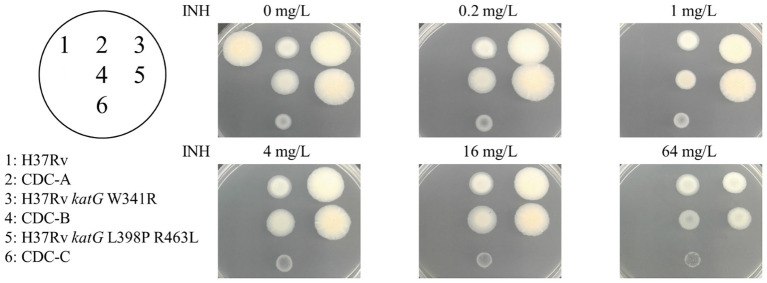
INH susceptibility testing of the control (H37Rv, spot 1), clinical isolates [CDC-A (spot 2), CDC-B (spot 4), and CDC-C (spot 6)] and H37Rv isogenic mutants [H37Rv *katG* W341R (spot 3), and H37Rv *katG* L398P R463L (spot 5)].

Three INH-R strains, CDC-A, CDC-B, and CDC-C, were provided by TCDC. The MICs of the three INH-R isolates were all >4 mg/L, which were initially determined using the Sensititre™ MYCOTB MIC Plate. To identify the uncommon mutations that conferred INH resistance in these isolates, we sequenced the frequent INH resistance hotspots of *katG*, *inhA*, and *oxyR*-*aphC* regions. The results demonstrated that the three isolates were carried wild-type *inhA*, *oxyR*, and *aphC* genes and had mutations in the *katG* gene resulting in the following amino acid changes: W341R (TGG/CGG), L398P (CTG/CCG), and R146P (CGG/CCG), respectively. All of them also harbored the R463L (CGG/CTG) change in KatG, and this residue change has been confirmed earlier as a polymorphism (Figure 1). Then, we further confirm the INH susceptibilities of all three isolates and revealed those were >64 mg/L by the agar dilution assay (Figure 2). The results indicate that the three amino acid residues change in *katG* might result in INH resistance in our INH-R isolates.

### H37Rv KatG-Complemented Clinical Strains Carrying W341R and L398P KatG Are More Susceptible to INH

**Figure 3:**
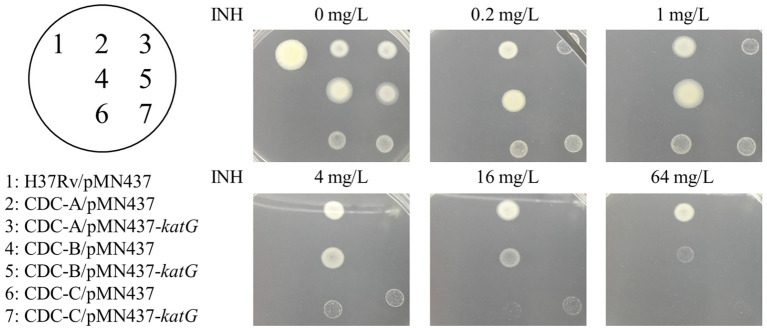
Complementation of INH resistance in the clinical isolates. INH susceptibility testing of the control H37Rv carrying the empty vector pMN437 (H37Rv/pMN437, spot 1); clinical isolate control strains carrying the empty vector pMN437 [CDC-A/pMN437 (spot 2), CDC-B/pMN437 (spot 4), and CDC-C/pMN437 (spot 6)]; and clinical isolates carrying wild-type *katG* expression plasmid [CDC-A/pMN437-*katG* (spot 3), CDC-B/pMN437-*katG* (spot 5), and CDC-C/pMN437-*katG* (spot 7)].

To delineate the association of the W341R, L398P, and R146P KatG proteins with the INH resistance in the isolates CDC-A, CDC-B, and CDC-C, we expressed the KatG protein from H37Rv in these INH-R strains using pMN437-*katG*. To examine whether expression of H37Rv KatG could restore INH resistance, INH susceptibility tests were conducted (Figure 3). The results showed that CDC-A/pMN437-*katG* was more susceptible to INH (MIC <4 mg/L) than the CDC-A carrying an empty vector (CDC-A/pMN437; MIC >64 mg/L). CDC-B/pMN437-*katG* was INH sensitive (MIC <0.2 mg/L), whereas CDC-B/pMN437 was highly resistant (MIC >64 mg/L). In contrast, CDC-C/pMN437-*katG* showed no difference in INH susceptibility when compared with CDC-C/pMN437 (Table 1). Therefore, we concluded that KatG W341R and L398P are the resistance-conferring amino acid changes in strains CDC-A and CDC-B, respectively. In contrast, KatG R146P is not the determinant for INH resistance of the CDC-C strain.

**Table 1:** INH sensitivity test results for *Mycobacterium tuberculosis* clinical isolates with and without H37Rv KatG expression plasmid.

Strains	INH MIC (mg/L)	Susceptibility
H37Rv/pMN437	<0.2	Sensitive
CDC-A/pMN437	>64	Resistant
CDC-A/pMN437-*katG*	≦4(>1)	Resistant
CDC-B/pMN437	>64	Resistant
CDC-B/pMN437-*katG*	<0.2	Sensitive
CDC-C/pMN437	>64	Resistant
CDC-C/pMN437-*katG*	>64	Resistant

INH, isoniazid; MIC, minimum inhibitory concentration.

### The W341R and L398P KatG Proteins Confer INH Resistance in *M. tuberculosis*

To directly confirm the contribution of the W341R and L398P KatG proteins on INH resistance, the *katG* gene of H37Rv was replaced with a segment of the DNA from the clinical isolates containing these *katG* mutations. The DNA sequencing of the H37Rv isogenic mutants H37Rv *katG* W341R and H37Rv *katG* L398P R463L showed that the *katG* of H37Rv was successfully substituted with the *katG* gene from CDC-A and CDC-B, respectively. The INH susceptibilities of the H37Rv isogenic mutants H37Rv *katG* W341R and H37Rv *katG* L398P R463L were measured by the agar dilution method (Figure 2), and revealed that, like CDC-A and CDC-B, both strains were resistant to the highest INH concentration tested (64 mg/L), indicating a MIC >64 mg/L and demonstrating that *katG* W341R and L398P confer INH resistance (Table 2).

**Table 2:** Drug susceptibility testing of clinical isolates and H37Rv isogenic mutants harboring mutant *katG*.

Strains	INH MIC (mg/L)	Susceptibility
H37Rv	<0.2	Sensitive
H37Rv *katG* W341R	>64	Resistant
H37Rv *katG* L398P R463L	>64	Resistant
CDC-A	>64	Resistant
CDC-B	>64	Resistant

INH, isoniazid; MIC, minimum inhibitory concentration.

## Discussions

INH is a first-line TB drug, and researchers have clarified various mechanisms of INH resistance by collecting INH-R strains and identifying the mutated genes. Studies have shown that 50–94% INH-R strains have at least one mutation in *katG*, 10–35% INH-R strains carry at least one mutation in the *inhA* promoter, and 10–40% INH-R strains carry at least one mutation in *oxyR*-*ahpC* region (Li et al., 2015). The *katG* 315 mutation, which leads to high-level INH resistance and the *inhA*-15 mutation, which leads to low-level INH resistance, are two of the most common mutations (Yao et al., 2010; Zenteno-Cuevas et al., 2019). However, mutations in the *oxyR*-*ahpC* region have not yet been recorded to confer INH resistance directly (Kandler et al., 2018). Studies reported that the expression level of alkylhydroperoxidase (AhpC) was different in INH-R strains with mutations in *katG*, indicating that AhpC were compensated for the loss of KatG activity and restored the anti-oxidative stress capacity (Ng et al., 2004; Nieto et al., 2016).

In this study, the KatG sequences of isolates CDC-A, CDC-B, and CDC-C revealed that each had a mutation at R463L (CGG/CTG) and had another mutation, which have not been previously reported as INH resistance-conferring mutations at W341R (TGG/CGG), L398P (CTG/CCG), and R146P (CGG/CCG), respectively. KatG R463L, was previously identified as a polymorphism, which is irrelevant to INH resistance (Johnsson et al., 1997; Ramaswamy et al., 2003; Lempens et al., 2018), and an epidemiological marker for Beijing strains (Tsolaki et al., 2005).

There are three studies, which had mentioned the mutations at KatG W341 in INH-R *M. tuberculosis*. A genetic analysis by next-generation sequencing performed in Ukraine. Daum et al. found a poly-resistant strain of *M. tuberculosis* containing the amino acid substitutions W341R and R463L in KatG (Daum et al., 2018), which are the same amino acid changes as strain CDC-A. A strain with MIC >1 mg/L found to have mutations at W341G and R463L by DNA sequencing (Brossier et al., 2006); and a group in Brazil found a strain with an INH MIC >32 mg/L harbors KatG W341S (Cardoso et al., 2004). In this study, the MIC of INH for H37Rv *katG* W341R confirmed that W341R is the INH resistance determinant in *M. tuberculosis*. Therefore, we concluded that amino acid residue 341 of KatG could be crucial for INH resistance in *M. tuberculosis*.

The INH resistance of strain CDC-C could not be reversed by expression of H37Rv KatG. Therefore, we concluded that the mechanism underlying INH resistance in the CDC-C strain may involve another INH-associated gene than those studied or other non-explored resistance mechanism.

The mutagenesis system described here could be adapted in future studies of drug resistance mechanisms in *M. tuberculosis* to establish a more reliable genetic diagnosis. Once the resistance-conferring mutation is identified, clinical institutions could shorten the time for drug susceptibility testing to facilitate the control and treatment of drug-resistant *M. tuberculosis*.

## Data Availability Statement

The original contributions presented in the study are included in the article/Supplementary Material, and further inquiries can be directed to the corresponding author.

## Author Contributions

J-TW performed experiment conception and design. W-HL, H-YT, W-TL, and RJ performed clinical strains analysis. L-YH and L-YL performed investigation. L-YH, L-YL, T-LL, and P-FH performed data analysis. L-YH, P-FH, and J-TW wrote manuscript. All authors contributed to the article and approved the submitted version.

## Conflict of Interest

The authors declare that the research was conducted in the absence of any commercial or financial relationships that could be construed as a potential conflict of interest.

## Supplementary Material

The Supplementary Material for this article can be found online at: https://www.frontiersin.org/articles/10.3389/fmicb.2020.01644/full#supplementary-material.

## Nomenclature

TB: Tuberculosis
INH: Isoniazid
INH-R: INH-resistant
WHO: World Health Organization
NAD^+^: Nicotinamide adenine dinucleotide
MICs: Minimum inhibitory concentrations
TCDC: Taiwan Centers for Disease Control
OADC: Oleic acid/albumin/dextrose/catalase
LB: Luria-Bertani
PCR: Polymerase chain reaction.
