# Supplementary material for: Two Novel katG Mutations Conferring Isoniazid Resistance in Mycobacterium tuberculosis
Source: Front Microbiol. 2020 Jul 15;11:1644. doi: 10.3389/fmicb.2020.01644 (PMC7374161; doi:10.3389/fmicb.2020.01644)
Supplement: Supplementary file 1 [file Data_Sheet_1.PDF]

Supplementary material

Table S1. Bacterial strains

**Strain**	**Characteristics**	**Source**
Mycobacterium tuberculosis		
H37Rv	Reference strain	ATCC 27294
H37Rv/pMN437	Protein expression control strain	This study
H37Rv KatG W341R	KatG W341R recombinant	This study
H37Rv KatG L398P R463L	KatG L398P R463L recombinant	This study
CDC-A	Clinical isolate; KatG W341R R463L	This study
CDC-A/pMN437	Protein expression control strain	This study
CDC-A/pMN437-*katG*	H37Rv KatG expression strain	This study
CDC-B	Clinical isolate; KatG L398P R463L	This study
CDC-B/pMN437	Protein expression control strain	This study
CDC-B/pMN437-*katG*	H37Rv KatG expression strain	This study
CDC-C	Clinical isolate; KatG R146P R463L	This study
CDC-C/pMN437	Protein expression control strain	This study
CDC-C/pMN437-*katG*	H37Rv KatG expression strain	This study
Escherichia coli		
DH10B	For plasmid construction	

Table S2. Primers used in the study

Primer	Sequence	Purpose	Source
katG F	GTCACACTTTCGGTAAGAC	*katG* sequence	(Jou et al., 2019)
katG R	TTGTCGCTACCACGGAACG	*katG* sequence	(Jou et al., 2019)
inhAr-F	AATTGCGCGGTCAGTTCCAC	*inhA* promoter sequence	(Jou et al., 2019)
inhAr-R	GTCGGTGACGTCACATTCGA	*inhA* promoter sequence	(Jou et al., 2019)
*inhA*1713-F	CCGAGGATGCGAGCTATATC	*inhA* sequence	(Jou et al., 2019)
*inhA*1713-R	GGCTCGGGTCGAAGTCCATG	*inhA* sequence	(Jou et al., 2019)
*inhA*2194-F	AGGCGCTGCTGCCGATCATG	*inhA* sequence	(Jou et al., 2019)
*inhA*2194-R	CCGAACGACAGCAGCAGGAC	*inhA* sequence	(Jou et al., 2019)
oxyR-ahpC-F	GCTTGATGTCGGAGAGCATCG	*oxyR-ahpC* sequence	(Jou et al., 2019)
oxyR-ahpC-R	GGTCGCGTAGGCAGTGCCCC	*oxyR-ahpC* sequence	(Jou et al., 2019)
katG GF	TCAGCGCACGTCGAACCTG	pMN437-*katG* insert amplification	This study
katG GR	GTGCCCGAGCAACACCCA	pMN437-*katG* insert amplification	This study
mycgfp2 inverse F	TATTAACCTCCTTTCTGTTAATTAAGC	pMN437 Vector reverse PCR	This study
mycgfp2 inverse R	ATTTAAATAAGCTTTAGCTAATTAATTGGG	pMN437 Vector reverse PCR	This study
katG OF	ACATTTCGGCGCCCTTTCTC	*katG* sequence and mutagenesis insert amplification	This study
katG OR	GACTGTGCTGTTGGCGAGG	*katG* sequence and mutagenesis insert amplification	This study
Amp-Sac1-F	AGAACGTTTTCCAATGATGAGCAC	*katG* mutagenesis construction	(Parish and Stoker, 2000)
Amp-Sac1-R	TCCTCCGATCGTTGTCAGAAGTAAG	*katG* mutagenesis construction	(Parish and Stoker, 2000)

katG OF2	TGTGGTTTCTTGCTGAGTGC	*katG* mutagenesis check integration	This study
katG OR2	TATACCGGACTACGCCGAAC	*katG* mutagenesis check integration	This study

Table S3. Plasmids

Plasmid	Characteristics	Source
pMN437	p_{smyc}-*mycgfp2*+; ColE1 origin; PAL5000 origin; Hyg^R;	(Song et al., 2008;Steinhauer et al., 2010)
pMN437-*katG*	pMN437-derived plasmid, containing *katG* gene from H37Rv, KatG expression vector	This study
pGOAL19	Hyg^R P_{Ag85}-lacZ P_{hsp60}-sacB PacI cassette vector, Amp^R	(Parish and Stoker, 2000)
pGOAL19-*katG* W341R R463L	pGOAL19-derived plasmid, containing *katG* gene from CDC-A	This study
pGOAL19-*katG* L398P R463L	pGOAL19-derived plasmid, containing *katG* gene from CDC-B	This study
